# High intensity exercise downregulates *FTO* mRNA expression during the early stages of recovery in young males and females

**DOI:** 10.1186/s12986-020-00489-1

**Published:** 2020-08-17

**Authors:** Jessica Danaher, Christos G. Stathis, Robin A. Wilson, Alba Moreno-Asso, R. Mark Wellard, Matthew B. Cooke

**Affiliations:** 1 School of Science, College of Science, Engineering and Health, RMIT University, Melbourne, Australia; 2 Institute for Health and Sport, Victoria University, Melbourne, Australia; 3 School of Medicine, NYU Langone Health, New York, USA; 4 Australian Institute for Musculoskeletal Science (AIMSS), Department of Medicine-Western Health, Melbourne Medical School, The University of Melbourne, Melbourne, Australia; 5 Science and Engineering Faculty, Queensland University of Technology, Brisbane, Australia; 6 Department of Health and Medical Sciences, Faculty of Health, Arts and Design, Swinburne University of Technology, Melbourne, VIC 3122 Australia

**Keywords:** FTO, Exercise, Skeletal muscle, Expression, Metabolomics

## Abstract

**Background:**

Physical exercise and activity status may modify the effect of the fat mass- and obesity-associated (*FTO*) genotype on body weight and obesity risk. To understand the interaction between FTO’s effect and physical activity, the present study investigated the effects of high and low intensity exercise on FTO mRNA and protein expression, and potential modifiers of exercise-induced changes in FTO in healthy-weighted individuals.

**Methods:**

Twenty-eight untrained males and females (25.4 ± 1.1 years; 73.1 ± 2.0 kg; 178.8 ± 1.4 cm; 39.0 ± 1.2 ml.kg.min^− 1^ VO_2peak_) were genotyped for the *FTO* rs9939609 (T > A) polymorphism and performed isocaloric (400 kcal) cycle ergometer exercise on two separate occasions at different intensities: 80% (High Intensity (HI)) and 40% (Low Intensity (LO)) VO_2peak_. Skeletal muscle biopsies (*vastus lateralis*) and blood samples were taken pre-exercise and following 10 and 90 mins passive recovery.

**Results:**

*FTO* mRNA expression was significantly decreased after HI intensity exercise (*p* = 0.003). No differences in basal and post-exercise FTO protein expression were evident between *FTO* genotypes. Phosphorylated adenosine monophosphate-activated protein kinase (AMPK) and Akt substrate of 160 kDa (AS160) were significantly increased following HI intensity exercise (*p* < 0.05). Multivariate models of metabolomic data (orthogonal two partial least squares discriminant analysis (O2PLS-DA)) were unable to detect any significant metabolic differences between genotypes with either exercise trial (*p* > 0.05). However, skeletal muscle glucose accumulation at 10 mins following HI (*p* = 0.021) and LO (*p* = 0.033) intensity exercise was greater in AA genotypes compared to TT genotypes.

**Conclusion:**

Our novel data provides preliminary evidence regarding the effects of exercise on FTO expression in skeletal muscle. Specifically, high intensity exercise downregulates expression of *FTO* mRNA and suggests that in addition to nutritional regulation, *FTO* could also be regulated by exercise.

**Trial registration:**

ACTRN12612001230842. Registered 21 November 2012 – Prospectively registered, https://www.anzctr.org.au/

## Background

The fat mass- and obesity-associated (*FTO*) gene was initially considered an “obesity gene” when early human studies demonstrated significant associations between its genetic polymorphism and body mass index (BMI) [1, 2]. Subsequent studies using transgenic overexpression and gene knockout models have sought to understand these associations by determining FTO’s biological role within various tissues [3–5]. While it is evident that FTO targets neural tissue and its energy homeostatic functions, whole body loss-of-function mutations may mask other tissue-specific activities attributed to FTO. Studies have shown that homozygous knockout of FTO leads to loss of lean mass [4, 5], which does not occur in targeted hypothalamic knockout models [5], suggesting FTO can promote its biological effects through pathways and tissues other than neural.

Skeletal muscle is the largest energy producing and consuming organ within the body and is influential on energy expenditure and whole body metabolism. Recent evidence suggests that FTO may act as an ‘energy sensor’ within skeletal muscle regulating important cellular metabolic pathways for substrate utilisation, storage and growth [6, 7]. This role could be via 5′ adenosine monophosphate-activated protein kinase (AMPK) regulation, as AMPK is recognized as a key energy sensor that can regulate energy status [8]. Activation of AMPK increases glucose uptake in skeletal muscle [9], enhances lipid oxidation [10] and reduces fatty acid incorporation into triacylglycerol [11]. Conversely, ablation of AMPK reduces fatty acid oxidation and enhances skeletal muscle lipid accumulation, leading to elevated triglyceride content [12]. Recent work by Wu and colleagues [7] suggests a relationship between FTO and AMPK and molecular regulation of lipid metabolism and metabolic diseases. Specifically, AMPK’s regulation of lipid accumulation in skeletal muscle could be via FTO-dependent demethylation of mRNA, with inhibition of AMPK upregulating FTO expression and enhancing lipid accumulation, while activation of AMPK downregulates FTO expression and reduces lipid accumulation [7].

Exercise is perhaps the most powerful physiological activator of AMPK [13]. When activated, AMPK stimulates energy generating processes such as glucose uptake and fatty acid oxidation and decreases energy consuming processes such as protein and lipid synthesis. In addition, AMPK activation in peripheral tissues seems to counteract many of the cellular abnormalities observed in animal models of metabolic syndrome including insulin resistance, inflammation and ectopic lipid deposition. These observations could explain in part how higher physical activity levels attenuate the influence of FTO variation on obesity risk [14], and why lifestyle interventions demonstrate greater efficacy in promoting weight loss in *FTO* risk A-allele carriers compared to those carrying the TT genotype [15]. It is possible that exercise-induced activation of AMPK downregulates FTO expression and/or leads to reduced FTO-dependent demethylation of mRNA which subsequently enhances lipid oxidation and reduces fat deposition. Over the long term this could result in weight loss. However, no study to date has looked at the effect of exercise on skeletal muscle FTO mRNA and protein levels and whether such effects are mediated via changes in AMPK. Additionally, using a metabolomics approach to analyse relative concentrations of multiple metabolites can help characterise core metabolic changes (i.e. metabolite “signature”) that may otherwise be missed when analysing single or multiple metabolite changes. Importantly, this technology can assist in identifying potential variations in metabolic pathways between genotypes which could assist in elucidating the mechanisms by which exercise regulates FTO.

Thus, the present study investigated the effect of exercise-induced metabolic perturbations on skeletal muscle FTO mRNA and protein expression, and determined whether these changes are genotype variant specific. In addition, this study sought to identify potential metabolic modifiers of FTO expression. It was hypothesized that higher exercise intensity would cause larger metabolic perturbations and AMPK activation, leading to greater downregulation of skeletal muscle *FTO* mRNA expression in variants encompassing the risk A-allele (AA and AT genotypes) compared to individuals homozygous for the non-risk allele (TT genotypes).

## Methods

### Participants

A total of 28, apparently healthy, sedentary males and females (25.4 ± 1.1 years; 73.1 ± 2.0 kg; 178.8 ± 1.4 cm; 39.0 ± 1.2 ml.kg.min^− 1^ peak oxygen uptake (VO_2peak_)) volunteered to take part in this study. Participants were excluded from participating if they had diagnosed diabetes (fasting blood glucose greater than 7.0 mmol. L^− 1^), were performing any regular fitness training (> 30 mins, 3 x per week) for 6 months prior, taking contraindicated prescription medication which influence metabolism (including thyroid, hyperlipidmeic, hypoglycemic, or antihypertensive), or were pregnant. Participants believed to meet the eligibility criteria were asked to provide written informed consent based on documents previously approved by the Victoria University Human Research Ethics Committee (HRETH 12/197) and all procedures were performed in accordance with the ethical standards set out in the 1964 Declaration of Helsinki.

### Preliminary testing

#### Genotyping

Prior to the experimental exercise trials, cells from inside each participant’s cheek were collected using a standard buccal swab, with QuickExtract solution (Illumina) used to extract DNA from these swabs. Genotyping of the rs9939609 (T > A) polymorphism of the *FTO* gene was performed using a Taqman allelic discrimination assay (Life Technologies, VIC, Australia) and a CFX96 Real-Time thermal cycler (Bio-Rad Laboratories, VIC, Australia) as per manufacturer’s instructions. For quality control purposes, a positive and negative control was used. The context sequence for the SNP tested was [VIC/FAM] GGTTCCTTGCGACTGCTGTGAATTT [A/T]GTGATGCACTTGGATAGTCTCTGTT. The overall genotyping efficiency was 100%.

#### Body composition assessment

Dual Energy X-ray Absorptiometry (DEXA; Hologic Discovery W, MA, USA) was used to assess body composition. Calibrations were performed the morning of DEXA analysis, and participants were in a standardised supine position throughout the duration of the scan. A whole-body scan was used (~ 1.5 mSv) to identify total body mass, fat mass, lean muscle mass and bone mineral content. Height, hip and waist circumference, and blood pressure were measured using a stadiometer, tape measure and sphygmomanometer (Omron HEM7322; Omron Healthcare, VIC, Australia), respectively.

#### Graded exercise test

To ascertain the fitness level of participants, VO_2peak_ was measured approximately one week prior to the first experimental exercise trial. A standard graded exercise protocol on an Excalibur Lode Cycle ergometer (Netherlands) was performed: Males, 3 × 3 min sub-maximal workloads at 50, 100 and 150 W followed by successive 1-min workload increments of 25 W until volitional exhaustion; Females, 3 × 3 min sub-maximal workloads at 25, 50 and 75 W followed by successive 1-min workload increments of 25 W until volitional exhaustion. Participants were encouraged to maintain a pedal frequency between 80 and 100 revolutions per minute (rpm) and the test was terminated when this could not be maintained for a period of 5 s. Expired air was directed by a Hans Rudolph valve via a ventilometer into a mixing chamber and analysed for oxygen and carbon dioxide content (Moxus; AEI Technologies, PA, USA). Prior to each VO_2peak_ test the gas analyser was calibrated using commercially prepared gas mixtures (BOC Gases, Australia). Data obtained from the graded exercise test were used to calculate the workload each participant required for the subsequent experimental exercise trials at 80 and 40% of their VO_2peak_.

### Experimental exercise trial protocol

Participants were asked to complete two isocaloric acute exercise trials in a non-randomised order, separated by at least 1 week for males and 1 month for females: i) High Intensity (HI), 80% VO_2peak_ (AA, 127.6 ± 13.1 W; AT, 126.0 ± 15.0 W; TT, 113.9 ± 15.1 W), and ii) Low Intensity (LO), 40% VO_2peak_ (AA, 63.8 ± 6.6 W; AT, 62.9 ± 7.5 W; TT, 57.1 ± 7.6 W). Similar to the VO_2peak_ test, these protocols were performed on an Excalibur Lode Cycle ergometer (Netherlands) and participants were encouraged to maintain a pedal frequency between 80 and 100 rpm. Exercise was stopped once each participant had expended 400 kcal as estimated via indirect calorimetry (Moxus; AEI Technologies, PA, USA). Substrate utilisation was calculated using standard stoichiometric equations [16], with the assumption that protein oxidation was minor and constant. Energy expenditure was calculated based on the following formula, with respiratory values in l.min^− 1^ units:

$$ \mathrm{Energy}\ \mathrm{Expenditure}\ \left(\mathrm{kJ}.{\min}^{-1}\right)=16.318\ast {\mathrm{VO}}_2-4.602\ast {\mathrm{VCO}}_2 $$

Respiratory Exchange Ratio (RER) data were used to examine the response to metabolic demand by measuring the area under the curve (AUC) for RER transition from the beginning to the end of exercise. Borg Scale Ratings of Perceived Exertion (RPE 6–20 scale) were recorded every 10 mins throughout the exercise bouts, and immediately upon cessation of exercise, to determine perceived physical demand between allelic variants of *FTO*.

Participants were asked to refrain from consuming caffeine and alcohol, and from undertaking strenuous exercise 24 h prior to attending the experimental exercise trials. Participants recorded their dietary intake for 24 h before the first experimental exercise trial and were asked to replicate meals the day prior to the subsequent trial. Experimental exercise trials were conducted in the morning, approximately 10–12 h after the last meal to produce basal state conditions. Exercise was preceded by a rest period and followed by 90 mins of passive recovery in a supine position.

### Plasma analysis

Each participant had an intravenous cannula inserted into a vein in the antecubital space to obtain blood samples throughout each experimental exercise protocol, and this was kept patent with isotonic saline (0.9% NaCl, Pfizer). Blood was sampled pre-exercise (0 mins), and at 10 and 90 mins throughout the post-exercise passive recovery period. Samples were immediately placed into lithium heparin (BD Vacutainer) tubes and centrifuged at 12,000 rpm for 2 min. Plasma samples were decanted and analysed for glucose concentration (YSI 2300 STAT; Yellow Springs Instruments, OH, USA). Plasma albumin was measured using a commercially available Bromocresol Green Albumin Assay Kit (Sigma Aldrich, Australia) and used as an indirect marker and estimation of plasma volume changes during exercise [17].

### Skeletal muscle analysis

#### Skeletal muscle biopsies

Skeletal muscle biopsies were collected from the *vastus lateralis* tissue under local anaesthesia pre-exercise (0 mins), and at 10 and 90 mins throughout the post-exercise passive recovery period. A fresh incision was made for each biopsy, which was taken distal to proximal (at least 1 cm apart) in the middle of the muscle belly, approximately 5–8 cm above the left kneecap. Muscle sampling was performed using a Bergström needle with suction [18]. Muscle samples were immediately snap-frozen in liquid nitrogen and stored at − 80 °C until analysis.

#### Metabolomics analysis – GC-MS

##### Muscle metabolite extraction and preparation

Approximately 20 mg wet weight of each skeletal muscle sample was diluted with 250 μl of methanol (MeOH) [spiked with 4% ^13^C_6_-Sorbitol as an extraction internal standard (ISTD)]. Supernatant from completely homogenized samples were separated and transferred into 6 mm diameter conical bottom glass vial inserts (Phenomenex, NSW, Australia). Pooled biological quality control (PBQC) samples were created using 10 μl of supernatant from each extracted sample. Samples were then dried in vacuo (RVC 2–33, John Morris, Australia) at a temperature of − 55 °C and pressure of 3 mbar for 3 h, prior to being placed into glass vials for Gas Chromatography-Mass Spectroscopy (GC-MS) analysis. Additional glass vials containing methyloxime (MeOX) (10 μl per sample) and trimethylsilane (TMS) (20 μl per sample) were prepared for derivatisation.

##### Instrumentation and data handling

The GC-MS system used comprised of a 7000B Agilent GC triple-quadrupole and a 5975C Agilent triple-axis MS detector (Agilent Technologies, CA, USA). A MPS2XL GC-MS autosampler (Gerstal Technologies, Mülheim, Germany) was set to select samples for analysis in a randomised order. MeOX and TMS derivatised samples were injected onto the GC column using a hot needle technique. The injection was operated in splitless (1 μl sample) and split (0.20 μl sample) modes to avoid overloaded chromatogram peaks. The instrumentation conditions and data handling procedures (including mass spectra and peak verification processes) were as previously described [19]. Overloaded peaks (lactate, glucose, mannose, sucrose, fructose, urea and cholesterol) were analysed separately from the split mode. Muscle metabolite concentrations (expressed as arbitrary units (AU)) for each metabolite detected in each sample were normalised to the ISTD (^13^C_6_-Sorbitol) and to muscle sample wet weight.

#### Skeletal muscle mRNA expression

Total RNA was isolated from ~ 20 mg skeletal muscle using TRIzol reagent. Total RNA concentration and purity of each sample was determined using a Nanodrop Spectrophotometer (Thermo Scientific, VIC, Australia). RNA (1 μg) was reverse transcribed to cDNA using an iScript cDNA Synthesis Kit (Bio-Rad Laboratories, VIC, Australia), as per manufacturer’s instructions. Relative mRNA expression was determined by QuantStudio 7 Flex (Applied Biosystems, CA, USA) using 20X PrimePCR Assays and SsoAdvanced Universal SYBR Green Supermix (Bio-Rad Laboratories, VIC, Australia). mRNA sequences of the oligonucleotide primers used are listed in Supplementary Table S-1. *ß-Actin* (*ACTB*) was used as an internal control standard for each reaction due to its previous verification as a constitutively expressed housekeeping gene in human skeletal muscle following acute exercise [20]. The relative amount of the target mRNA was calculated using the fold change 2^-∆∆CT^ method [21].

#### Skeletal muscle protein expression

Twenty-five cryosections of skeletal muscle (30 μm sections) were lysed in homogenisation buffer (0.125 M Tris HCl, 10% Glycerol, 4% SDS, 10 mM EGTA, 0.1 M DTT [pH 8.0]). Total protein concentration of muscle lysate was measured using Pierce BCA protein estimation (Abcam, VIC, Australia) and RED 660 Protein Assay with SDS neutralizer (G Bioscience, MO, USA), as per manufacturer’s instructions. Protein was resolved on 7.5% or 12% Mini-PROTEAN TGX Stain-Free Gels (Bio-Rad Laboratories, VIC, Australia) and transferred to PVDF membrane. Membranes were blocked with skim milk in Tris-Buffered Saline-Tween (TBST) and incubated overnight with the following primary antibodies: FTO (GeneTex #GTX63821), pan Actin (NeoMakers #MS-1295-P0), Phospho Ser588 AS160 (Akt substrate of 160 kDa) (Cell Signalling Technology (CST) #8730), total AS160 (CST #2447), Phospho AMPKα (CST #2535) and total AMPKα (CST #2603). After incubation, membranes were washed and incubated for 1 h at 4 °C with horseradish peroxidase-linked secondary antibody (CST #7074). Proteins were detected via chemiluminescence using Clarity Western ECL Substrate within a VersaDoc Imager (Bio-Rad Laboratories, VIC, Australia). Densitometry was performed using Image Lab Software (Bio-Rad Laboratories, VIC, Australia) with the total proteins in each lane of the stain free PVDF membrane normalised to internal controls run on each gel. FTO content was expressed relative to Actin, phosphorylated AS160 content was expressed relative to total AS160, and phosphorylated AMPK was reported relative to total AMPK.

### Data analysis and statistical methods

#### Sample size calculation

The estimated sample size for the main outcome measures of gene expression was based on an assumed correlation of 0.7 between the pre and post exercise outcome measures, and an effect size of Cohen’s d = 0.62 for fold change in skeletal muscle mRNA expression of metabolic genes following acute high and/or low intensity exercise [22, 23]. In order to have power of 80% and a significance level of 5%, an estimated total sample size of 28, taking into account a predicted ~ 20% dropout rate.

#### Multivariate analysis

An orthogonal two partial least squares discriminant analysis (O2PLS-DA) multivariate model was used as an analogous extension of the common PLS-DA model. This multivariate analysis model was selected due to its previously shown suitability in combining ‘omics’ data [24]. Model quality was reported for O2PLS-DA using R^2^X(cum) and Q^2^, which represents, the measure of fit (i.e. the explained variation in metabolites) and the goodness of prediction (i.e. the variation in genotype that can be predicted by the model), respectively, as estimated by cross-validation (SIMCA statistical modelling, version 14, MKS, Sweden). The maximum possible Q^2^ value is 1.0 as it is a fraction of the total variability, therefore Q^2^ ≥ 0.7 can be considered a good predictor and < 0.5 as insignificant. Likewise, the maximum possible R^2^X(cum) value is 1.0, with this representative of a perfectly fitting model, whilst a R^2^X(cum) value of 0.0 would indicate no model fit at all. The area under the curve (AUC) of the receiver operating characteristic (ROC) curve was used to determine the overall accuracy and separation performance of the genotypes in each O2PLS-DA model. MetaboAnalyst 3.0 [25] was used to generate an additional PLS-DA on the whole set of metabolites (variables) at each time point, with normalisation via a generalised log transformation applied to the data matrix to improve symmetry. This multivariate pattern analysis model determined the metabolites with variable importance for projection (VIP) values ≥1.0. The VIP value was used to reflect variable importance, and the metabolite subset with values ≥1.0 is herein referred to as ‘VIP metabolites’.

#### Univariate analysis

VIP metabolites were selected for further analysis (to reduce variability) and analysed using SPSS software (IBM SPSS Statistics for Windows, Version 20, NY, USA). Unpaired two-way ANOVA’s with repeated-measures were used to calculate individual significance for each genotype with time as the within group factor and genotype as the between group factor. Where univariate analysis revealed any significant main effects for time, subsequent pairwise comparisons were performed to detect differences over time. Where a genotype by time interaction was detected, multiple comparisons with Tukey’s post hoc tests were completed to identify differences. One-way ANOVA’s were performed for participant characteristic and indirect calorimetry (substrate utilisation, energy expenditure and RER) data, with unpaired t-tests completed when interactions between factors were found. Linear regression and covariant analysis (ANCOVA) were used to determine the effect of age and sex (both known to influence associations between *FTO* rs9939609 and obesity-related traits) on allelic representation of dependent variables. Regression analysis was used to observe relationships between skeletal muscle mRNA expression of metabolic genes and muscle metabolites showing genotype by time interactions. Data are expressed as mean ± SEM unless otherwise stated. The level of significance was set at *p* < 0.05.

## Results

### Participant characteristics

Participant characteristics for each *FTO* rs9939609 genotype were similar with no significant differences in total body mass, height, BMI, hip and waist circumference, fat mass, lean muscle mass, bone mineral content, blood pressure or VO_2peak_ noted (*p* > 0.05) (Table 1). A genotype effect was detected for age (*p* = 0.038), with AT genotypes significantly older than TT genotypes (*p* = 0.019). A trend towards significance for a genotype effect was detected for fasting plasma glucose concentrations (*p* = 0.057).

**Table 1:** Participant characteristics when separated by *FTO* genotype of the rs9939609 polymorphism

	Genotype			
	AA	AT	TT	***P*** value
n	10	9	9	/
Sex	5F / 5 M	4F / 5 M	6F / 3 M	/
Age (yr)	24.4 ± 1.7	29.3 ± 2.2	22.7 ± 1.2	**0.038**
Total Body Mass (kg)	74.5 ± 3.9	72.0 ± 2.6	72.6 ± 4.2	0.875
Height (cm)	176.1 ± 2.8	169.9 ± 2.4	172.1 ± 1.9	0.212
BMI (kg/m^2^)	24.0 ± 1.0	25.0 ± 1.1	24.4 ± 1.1	0.791
Hip Circumference (cm)	99.6 ± 2.4	99.6 ± 2.4	101.4 ± 1.8	0.813
Waist Circumference (cm)	81.8 ± 3.8	79.6 ± 2.8	77.8 ± 3.0	0.674
Fat Mass (%)	24.9 ± 1.6	22.6 ± 3.3	27.2 ± 2.0	0.416
Fat Mass (kg)	18.1 ± 1.0	16.1 ± 2.7	19.6 ± 2.0	0.486
Lean Muscle Mass (kg)	53.4 ± 3.5	52.3 ± 2.7	52.1 ± 3.3	0.689
Bone Mineral Content (kg)	2.6 ± 0.1	2.5 ± 0.1	2.5 ± 0.1	0.737
Systolic BP (mmHg)	129.0 ± 5.1	128.7 ± 4.2	122.7 ± 1.9	0.480
Diastolic BP (mmHg)	76.4 ± 3.2	75.8 ± 2.5	75.1 ± 3.0	0.952
Fasting Plasma Glucose (mmol. L^− 1^)	5.0 ± 0.1	5.4 ± 0.1	4.9 ± 0.1	0.057
VO_2peak_ (ml.kg.min^− 1^)	40.0 ± 1.4	39.0 ± 2.6	37.9 ± 2.3	0.771

Values are expressed as mean ± SEM. *F* female, *M* male, *VO*_*2peak*_ peak oxygen uptake

### Participant’s physiological responses to HI and LO exercise trials

Workloads (W) performed during the HI and LO intensity exercise protocols were similar between genotypes (*p* > 0.05) (Table 2). Both HI and LO intensity exercise trials elicited an increase in heart rate, with higher elevations during the HI trial compared to the LO trial (data not shown). Heart rate was similar between genotypes before, during and following HI and LO intensity exercise within each trial (data not shown). Additionally, RPE (considered on a numerical scale and presented as median (interquartile range)) was similar between genotypes at the completion of each exercise protocol (HI: AA, 16 (14–17), AT 16 (15–18), TT, 17 (15–19) (representing “Hard – Very Hard”) (*p* = 0.254); LO: AA, 12 (11–13), AT 11 (11–12), TT, 13 (11–14) (representing “Fairly Light – Somewhat Hard”) (*p* = 0.456)). There were no significant differences between genotypes in time to expend 400 kcal during the HI (*p* = 0.511) and LO intensity (*p* = 0.472) exercise protocol, or for average RER (HI, *p =* 0.323; LO, *p =* 0.603), glucose utilisation (g.kgLBM^− 1^.T.I^− 1^) (HI, *p =* 0.740; LO, *p =* 0.310) and fat utilisation (g.kgLBM^− 1^.T.I^− 1^) (HI, *p =* 0.709; LO, *p =* 0.498) measured during each exercise protocol (Table 2). The transition in substrate utilisation during exercise (RER AUC) was not significantly different between genotypes for each exercise protocol (HI, *p =* 0.206; LO, *p =* 0.410) (Table 2). The absence of an effect of age or sex on respiratory gas exchange measurements between *FTO* genotypes was confirmed by ANCOVA (*p* > 0.05).

**Table 2:** Respiratory gas exchange measurements, and calculated fat and glucose utilisation, between *FTO* rs9939609 genotypes after isocaloric HI (80% VO_2peak_) and LO (40% VO_2peak_) intensity exercise

	Genotype			***P*** value
	AA	AT	TT	
**HI**				
Workload (W)	127.6 ± 13.1	126.0 ± 7.5	113.9 ± 7.6	0.767
T.I (min:sec)	36:45 ± 2:00	39:29 ± 3:07	41:21 ± 3:02	0.511
Av. VO_2_ (ml.kgbw.min^−1^)	29.0 ± 1.4	28.7 ± 2.1	27.0 ± 1.7	0.674
Av. RER	0.96 ± 0.07	0.99 ± 0.02	0.96 ± 0.01	0.323
RER AUC	62.7 ± 4.4	58.9 ± 5.8	71.9 ± 5.0	0.206
Fat utilisation (g.kgLBM^− 1^.T.I^− 1^)	0.12 ± 0.04	0.10 ± 0.04	0.14 ± 0.03	0.709
Glucose utilisation (g.kgLBM^− 1^.T.I^− 1^)	1.58 ± 0.13	1.68 ± 0.04	1.69 ± 0.12	0.740
Fat utilisation (%)	14.5 ± 4.5	10.2 ± 3.8	15.8 ± 3.2	0.593
Glucose utilisation (%)	85.5 ± 4.5	89.8 ± 3.8	84.2 ± 3.2	0.593
**LO**				
Workload (W)	63.8 ± 6.6	62.9 ± 7.5	57.1 ± 7.6	0.779
T.I (min:sec)	54:28 ± 2:58	57:59 ± 4:06	61:04 ± 4:19	0.472
Av. VO_2_ (ml.kgbw.min^− 1^)	20.0 ± 0.7	21.3 ± 1.2	19.0 ± 0.9	0.289
Av. RER	0.89 ± 0.01	0.91 ± 0.02	0.90 ± 0.01	0.603
RER AUC	82.6 ± 3.4	77.8 ± 6.5	86.4 ± 6.5	0.410
Fat utilisation (g.kgLBM^− 1^.T.I^− 1^)	0.31 ± 0.04	0.24 ± 0.05	0.32 ± 0.05	0.498
Glucose utilisation (g.kgLBM^− 1^.T.I^− 1^)	1.15 ± 0.08	1.33 ± 0.08	1.29 ± 0.10	0.310
Fat utilisation (%)	36.6 ± 4.0	30.1 ± 5.6	34.5 ± 4.3	0.607
Glucose utilisation (%)	56.4 ± 5.5	69.9 ± 5.6	57.2 ± 6.2	0.206

Values expressed as mean ± SEM. *AUC* area under the curve, *LBM* lean body mass, *Av. RER* average respiratory exchange ratio, *T. I* Time interval required to expend 400 kcal, *Av. VO*_*2*_ average oxygen uptake

### Metabolite analysis

#### Skeletal muscle metabolites: multivariate analysis

**Fig. 1:**
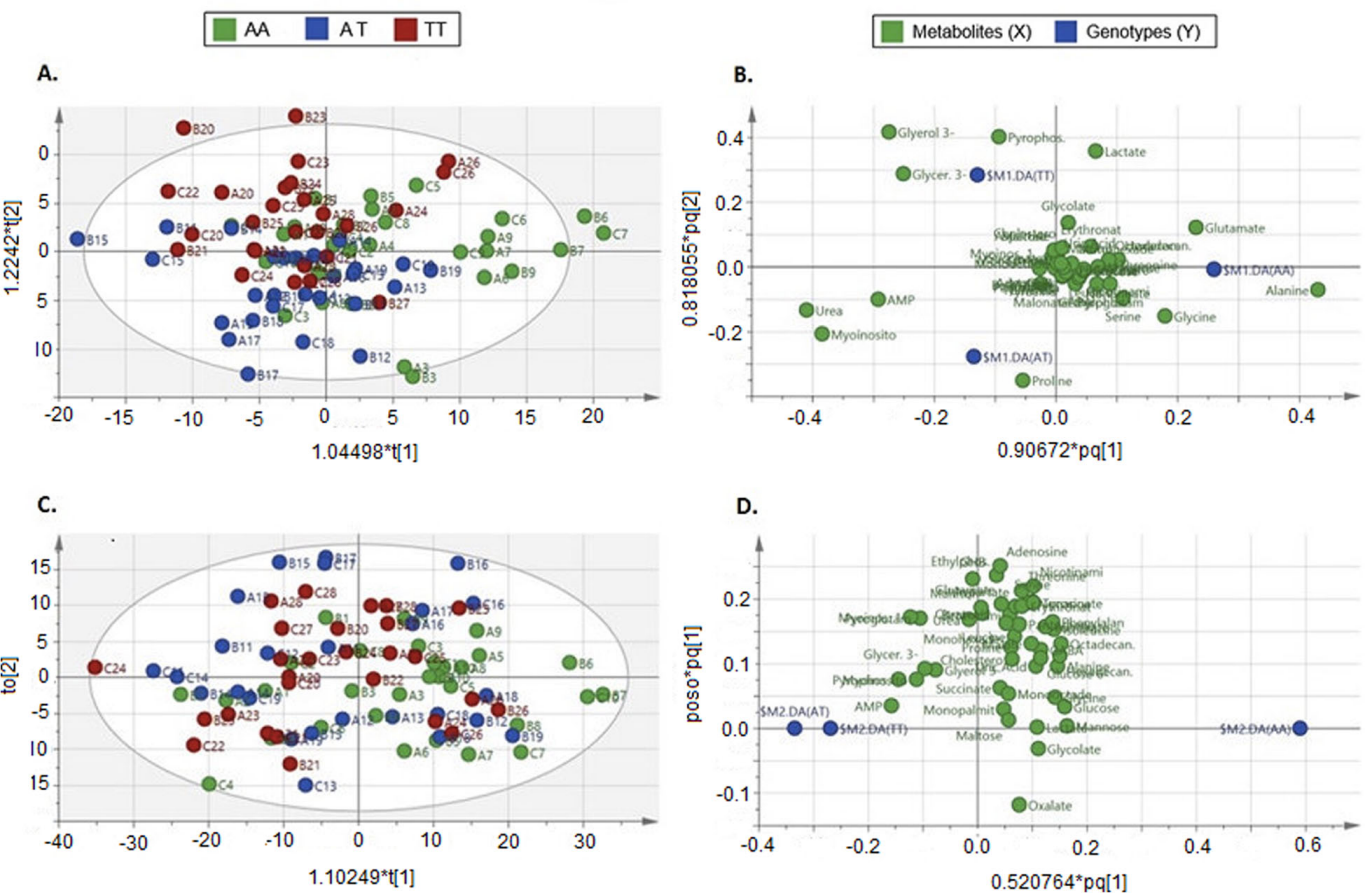
O2PLS-DA models of genotype variation for the **a** HI and **c** LO intensity exercise trials. O2PLS-DA models, **a** & **c** Green represent those homozygous for the risk A-allele (AA genotypes), blue is representative of heterozygous participants (AT genotypes), and red represents those who had not inherited the risk A-allele (TT genotypes). The ellipse is representative of a 95% confidence interval. Component 1 describes the orthogonal metabolite variation (within group variation) and Component 2 shows the primary variation between genotypes. Components are scaled proportionally to R^2^X (A, R^2^X[1] = 0.099, R^2^X[2] = 0.052; B, R^2^X[1] = 0.346, R^2^X[2] = 0.092). Loading plots of the O2PLS-DA models, **b** & **d** Genotype shown in blue and metabolites shown in green

Analysis of the chromatogram resulted in the detection of 48 identifiable metabolites (see Supplementary Table S-2 for metabolite identification details). Unpaired multivariate data models, O2PLS-DA with Pareto data scaling, were used to determine participant variation during the HI and LO intensity exercise trials, regardless of time (Fig. 1a & c). The O2PLS-DA modelling method demonstrated a similar metabolic signature between genotypes in the HI intensity exercise trial (*p* = 0.999), with very good validation metrics for data goodness of fit, R^2^X(cum) = 0.914, and very poor validation metrics for goodness of prediction, Q^2^ = 0.084 (Fig. 1a). Orthogonal variation in metabolites (X) accounted for 44% of the variation, and orthogonal variation between genotypes (Y) accounted for 32% of the variation. The O2PLS-DA modelling method also demonstrated a similar metabolic signature between genotypes in the LO intensity exercise trial (*p* = 0.982), with moderate validation metrics for data goodness of fit, R^2^X(cum) = 0.511, and very poor validation metrics for goodness of prediction, Q^2^ = 0.017 (Fig. 1c). Orthogonal variation in metabolites (X) accounted for 27% of the variation, and orthogonal variation between genotypes (Y) accounted for 17% of the variation.

The metabolites associated with each genotype can be extracted from the loadings scatter plot (Fig. 1b & d). Distribution of metabolites in the direction of each genotype signifies their contribution to model variation due to the respective genotype, whilst metabolites with the least importance are clustered in the centre. The metabolites likely to contribute most to each genotype in the HI intensity exercise trial model were, AA: alanine, glutamate and glycine; AT: proline, adenosine monophosphate (AMP), urea and myoinositol; TT: glycerol-3-phosphate (glyercol-3-P), glycerate-3-phosphate (glycerate-3-P) and pyrophosphate. Data from the LO intensity exercise trial did not provide sufficient power to differentiate metabolite variation in relation to genotype using loading plot observations, or to generate a secondary predictive component. AUC of the ROC curve showed a poorer fit in the LO intensity exercise trial compared to HI intensity exercise trial, with the AA genotype better described by the model than the TT genotype (see Supplementary Figure S-1).

Correlation coefficient scores based on the weighted sum of the PLS regression were used to identify the top 10 metabolites with the greatest influence on the components at each time point, regardless of exercise intensity (Supplementary Figure S-2). PLS-DA cross validation determined 27 metabolites in total with VIP scores ≥1. These VIP metabolites were used for subsequent univariate analysis to determine metabolic changes over time and between genotypes for the HI and LO intensity exercise trials.

#### Skeletal muscle metabolites: Univariate analysis

##### HI intensity exercise

A significant main effect for time was observed for skeletal muscle alanine, erythronate, fumarate, gamma hydroxybutyric acid (GHB), glucose, glutamate, glycine, glycolate, lactate, leucine, malate, maltose, mannose, monopalmitoglycerol, nicotinamide, phenylalanine, proline, tyrosine and uric acid following HI intensity exercise (*p* < 0.05) (see Supplementary Table S-3). Time as a main effect approached significance for muscle ß-alanine (*p* = 0.052) and glycerate-3-P (*p* = 0.056) following HI intensity exercise. At 10 mins post HI intensity exercise, muscle alanine, erythronate, fumarate, GHB, glucose, glycolate, lactate, malate, maltose, mannose, monopalmitoglycerol and tyrosine were significantly elevated compared to pre-exercise (*p* < 0.05), whereas muscle glutamate and proline were significantly decreased (*p* < 0.05). A trend for lower muscle nicotinamide was detected at 10 mins post HI intensity exercise compared to pre-exercise (*p* = 0.065). At 90 mins post HI intensity exercise, muscle erythronate and maltose were significantly elevated compared to pre-exercise (*p* < 0.05), with a trend towards significance for higher levels for glucose (*p* = 0.066), glycolate (*p* = 0.089) and uric acid (*p* = 0.060). Conversely, muscle fumarate, glutamate, glycine, leucine, phenylalanine and proline were significantly lower at 90 mins post HI intensity exercise compared to pre-exercise (*p* < 0.05). No main effect for genotype was identified for any of the VIP muscle metabolites (*p* > 0.05). A significant genotype by time interaction was observed for muscle glucose (*p* = 0.036), with subsequent analysis revealing a significantly higher level of muscle glucose in AA genotypes compared to TT genotypes at 10 mins following HI intensity exercise (*p* = 0.021).

##### LO intensity exercise

A significant main effect for time was observed for skeletal muscle alanine, erythronate, fumarate, glucose, glutamate, glycolate, glycerate-3-P, lactate, malate, maltose, monopalmitoglycerol, pyrophosphate and tyrosine following LO intensity exercise (*p* < 0.05) (see Supplementary Table S-3). Time as a main effect approached significance for muscle mannose (*p* = 0.068), uric acid (*p* = 0.074) and phenylalanine (*p* = 0.086). At 10 mins post LO intensity exercise, muscle alanine, erythronate, fumarate, glucose, glycolate, lactate, malate, monopalmitoglycerol and tyrosine were significantly elevated compared to pre-exercise (*p* < 0.05), with a trend for elevated muscle maltose (*p* = 0.060). At 90 mins post LO intensity exercise, muscle erythronate, glutamate, glycerate-3-P, glycolate, lactate, monopalmitoglycerol and pyrophosphate were significantly elevated compared to pre-exercise (*p* < 0.05), while only a trend towards significance for elevated fumarate (*p* = 0.064), maltose (*p* = 0.068) and glucose (*p* = 0.074) were observed compared to pre-exercise. No main effect for genotype was identified for any of the VIP muscle metabolites (*p* > 0.05). Similar to the HI intensity exercise trial, a genotype by time interaction was observed for muscle glucose (*p* = 0.035), with subsequent analysis revealing a significantly higher level of muscle glucose in AA genotypes compared to AT (*p* = 0.028) and TT (*p* = 0.033) genotypes at 10 mins post LO intensity exercise.

The absence of an effect of age or sex on muscle metabolite responses between *FTO* genotypes for both the HI and LO exercise trial was confirmed by ANCOVA (*p* > 0.05).

#### Plasma metabolites: Univariate analysis

**Fig. 2:**
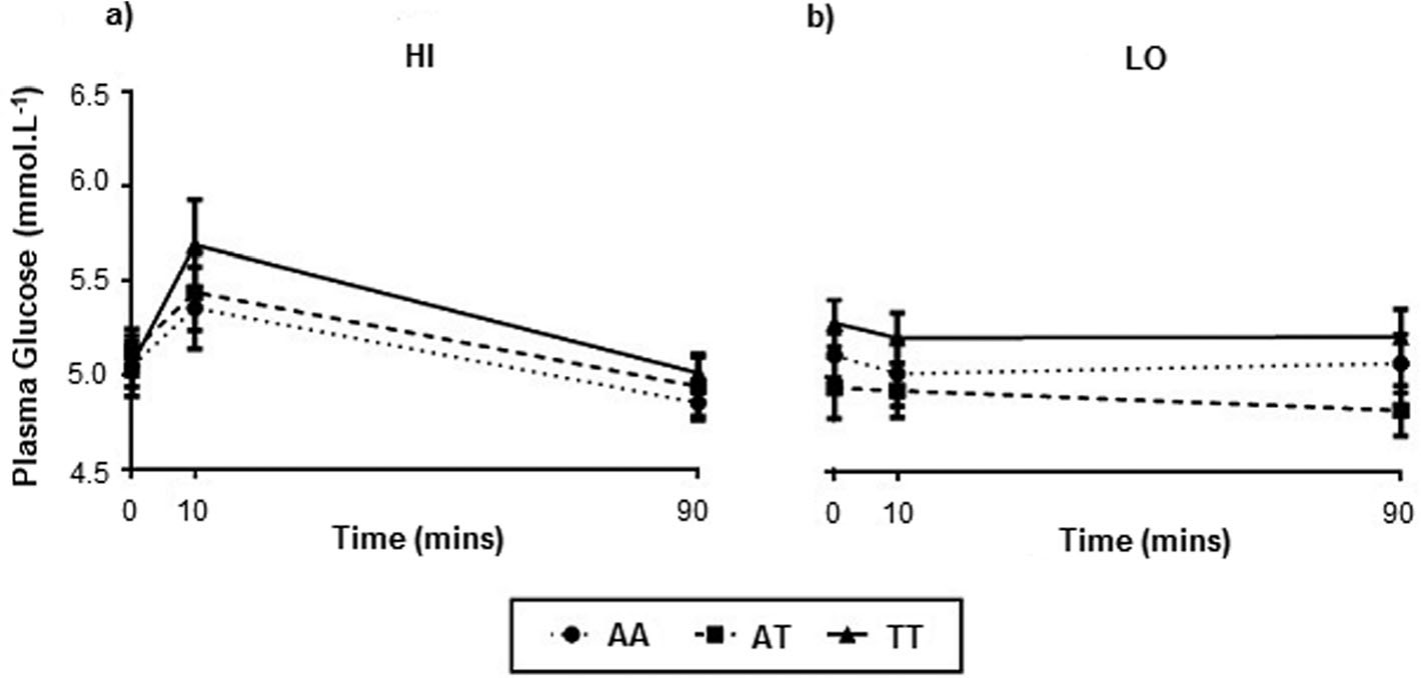
Plasma glucose concentrations between genotypes of the *FTO* rs9939609 polymorphism. Plasma glucose concentrations sampled prior to and following (during passive recovery) isocalorically matched HI (80% VO_2peak_) and LO (40% VO_2peak_) intensity exercise. Values expressed as mean ± SEM

Exercise-induced changes in plasma albumin concentrations were similar between genotypes at all observed time points during both exercise trials (data not shown) (*p* > 0.05). A significant main effect for time for plasma glucose was observed in the HI intensity exercise trial (*p* < 0.001). Subsequent pairwise comparisons revealed significantly higher plasma glucose at 10 mins (*p* = 0.001) and 90 mins (*p* = 0.042) post HI intensity exercise compared to pre-exercise (Fig. 2a). No main effect for time for plasma glucose was detected in the LO intensity exercise trial (*p* = 0.533) (Fig. 2b). No genotype main effect (HI, *p* = 0.656; LO, *p* = 0.196), or genotype by time interaction (HI, *p* = 0.681; LO, *p* = 0.932) was identified for either exercise trial.

### Skeletal muscle mRNA expression analysis

#### HI intensity exercise

**Fig. 3:**
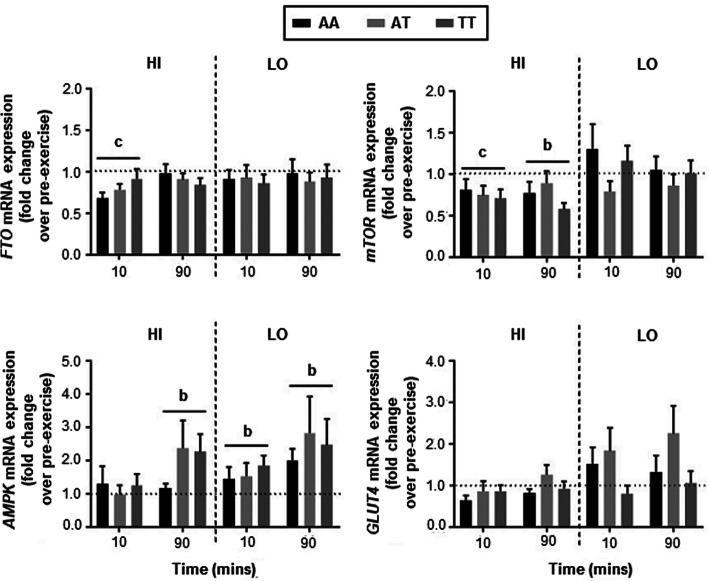
mRNA expression in human *vastus lateralis* skeletal muscle. Data are expressed as mean ± SEM of mRNA expression fold change over pre-exercise (set arbitrarily to 1) when normalised to *β-Actin* at 10 and 90 mins following HI (80% VO_2peak_) and LO (40% VO_2peak_) intensity exercise for *FTO*, *AMPK*, *GLUT4* and *mTOR*. Significant changes over time (from pre-exercise) represented by b = *p* < 0.01, and c = *p* < 0.001

A significant main effect for time was observed for *FTO* (*p* = 0.002), *AMPK* (*p* = 0.009) and *mTOR* (mammalian target of rapamycin) (*p* = 0.001) mRNA expression following HI intensity exercise at 80% VO_2peak_ (Fig. 3). Time as a main effect approached significance for *GLUT4* (glucose transporter type 4) mRNA expression (*p* = 0.054). Subsequent pairwise comparisons revealed a significant decrease in *FTO* (*p* < 0.001) and *mTOR* (*p* = 0.001) mRNA expression from pre-exercise to 10 mins post-exercise, and in *mTOR* (*p* = 0.002) from pre-exercise to 90 mins post-exercise. A significant increase in *AMPK* mRNA expression was observed from pre-exercise to 90 mins post-exercise (*p* = 0.009).

A weak trend for a genotype by time interaction was observed for *FTO* (*p* = 0.095). No genotype by time interactions were identified for the mRNA expression of *AMPK* (*p* = 0.304), *GLUT4* (*p* = 0.366) or *mTOR* (*p* = 0.377). No genotype main effects were identified for *FTO* (*p* = 0.894), *AMPK* (*p* = 0.606), *GLUT4* (*p* = 0.310) or *mTOR* (*p* = 0.611) mRNA expression. The absence of an effect of age or sex on *FTO* mRNA expression during the HI intensity exercise trial was confirmed by ANCOVA (*p* > 0.05).

#### LO intensity exercise

A significant main effect for time was observed for *AMPK* mRNA expression following LO intensity exercise at 40% VO_2peak_ (*p* = 0.009), with subsequent pairwise comparisons revealing a significance increase in *AMPK* mRNA from pre-exercise to 10 mins post-exercise (*p* = 0.005) and from pre-exercise to 90 mins post-exercise (*p* = 0.004) (Fig. 3). Time as a main effect approached significance for *GLUT4* (*p* = 0.093) mRNA expression following LO intensity exercise, with no main effect for time observed for *FTO* (*p* = 0.505) or *mTOR* (*p* = 0.642) mRNA expression.

No genotype main effects (*FTO*, *p* = 0.931; *AMPK*, *p* = 0.804; *GLUT4*, *p* = 0.164; *mTOR*, *p* = 0.280), or genotype by time interactions (*FTO*, *p* = 0.970; *AMPK*, *p* = 0.803; *GLUT4*, *p* = 0.277; *mTOR*, *p* = 0.528) were identified. The absence of an effect of age or sex on *FTO* mRNA expression during the LO intensity exercise trial was confirmed by ANCOVA (*p* > 0.05).

#### Regression analysis of mRNA

Regression analysis was used to determine if a relationship between skeletal muscle *FTO* mRNA and muscle glucose existed, as glucose was the only metabolite to demonstrate a genotype by time interaction. Further regression analyses between *FTO* mRNA and mRNA of other metabolic genes are in Supplementary Figure S-3.

A negative correlation was detected between skeletal muscle glucose levels and mRNA expression of *FTO* during the HI (*r* = − 0.234, *p* = 0.033) and LO intensity exercise trial (*r* = − 0.264 *p* = 0.017), regardless of time and genotype (see Supplementary Figure S-4). When accounting for genotype, the negative correlation remained between skeletal muscle glucose levels and mRNA expression of *FTO* in AA genotypes (HI, *r* = − 0.370, *p* = 0.044; LO, *r* = − 0.395, *p* = 0.031), during both exercise trials. No relationship between skeletal muscle glucose levels and mRNA expression of *FTO* was observed in AT genotypes (HI, *r* = − 0.205, *p* = 0.306; LO, *r* = − 0.291, *p* = 0.141) or TT genotypes (HI, *r* = − 0.100, *p* = 0.621; LO, *r* = 0.027, *p* = 0.899), during either the HI or LO intensity exercise trials. Further regression analyses between mRNA of other metabolic genes (*AMPK*, *mTOR* and *GLUT4*) and muscle glucose are in Supplementary Figure S-4.

### Skeletal muscle protein expression analysis

**Fig. 4:**
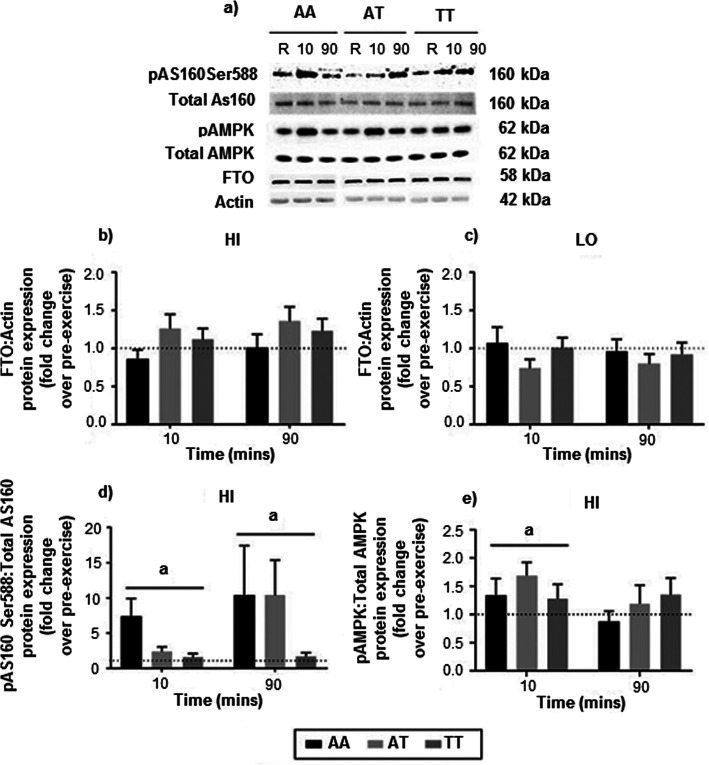
Expression levels of FTO, AS160 Ser588 phosphorylation, and phosphorylated AMPK in the *vastus lateralis* muscle. **a** Representative Western Blots of pAS160Ser588, total AS160, pAMPK, total AMPK, FTO and Actin for each genotype measured during pre-exercise rest (R), and at 10 mins and 90 mins during passive recovery following HI intensity exercise. **b** & **c** Relative expression of FTO protein when normalised to Actin following HI and LO intensity exercise respectively. **d** Relative expression of AS160 Ser588 phosphorylation (pAS160Ser588) relative to total AS160 following HI intensity exercise. **e** Relative expression of phosphorylated AMPK relative to total AMPK following HI intensity exercise. Data are expressed as mean ± SEM of the fold change at 10 and 90 mins compared to pre-exercise measurements (set arbitrarily to 1). ‘a’ represents significant main effect for time (*p* < 0.05)

No main effect for time (*p* = 0.128), genotype main effect (*p* = 0.181), or genotype by time interaction (*p* = 0.485) was detected for skeletal muscle FTO protein expression in response to HI intensity exercise (Fig. 4b). Similarly, LO intensity exercise did not have a significant effect on skeletal muscle FTO protein expression, with no main effect for time (*p* = 0.544), genotype main effect (*p* = 0.378) or genotype by time interaction (*p* = 0.650) observed (Fig. 4c).

A significant main effect for time was observed for pAS160Ser588 (*p* = 0.049; Fig. 4d) and pAMPK (*p* = 0.035; Fig. 4e) in skeletal muscle following HI intensity exercise. Subsequent pairwise comparisons revealed a significant increase in pAMPK relative to total AMPK from pre-exercise to 10 mins (*p* = 0.010) post-exercise, and a significant increase in pAS160Ser588 relative to total AS160 from pre-exercise to 10 mins (*p* = 0.011) and 90 mins (*p* = 0.046) post-exercise. No genotype main effects (pAMPK, *p* = 0.563; pAS160Ser588, *p* = 0.252), or genotype by time interactions (pAMPK, *p* = 0.490; pAS160Ser588, *p* = 0.386) were identified.

The absence of an effect of age or sex on FTO and pAMPK protein expression between *FTO* genotypes for both the HI and LO exercise trial was confirmed by ANCOVA (*p* > 0.05). However, there was a significant sex effect for pAS160Ser588, with females demonstrating a greater increase at 10 min recovery (*p* = 0.042) compared to males. No effect of age was evident. No relationships were detected between skeletal muscle levels of pAS160Ser588, pAMPK or FTO expression and the mRNA expression of *FTO*, *AMPK*, *mTOR* or *GLUT4* during the exercise trials, regardless of time and genotype (see Supplementary Figure S-5).

## Discussion

The present study provides a potential mechanism by which exercise may attenuate the influence of the *FTO* rs9939609 polymorphism on obesity risk. To the best of our knowledge, this is the first preliminary report on the effects of two isocaloric bouts of high and low intensity exercise on skeletal muscle *FTO* gene expression. The current findings demonstrate that an acute bout of high intensity exercise significantly downregulates skeletal muscle *FTO* mRNA during the early stages of recovery. This was not observed for lower intensity exercise. Downregulation of *FTO* mRNA expression was associated with elevated muscle glucose levels, but only in those individuals carrying the at risk AA genotype. Despite higher intensity exercise inducing greater metabolic perturbations compared to the low intensity trial, metabolomics analysis was unable to identify any unique metabolic differences between the *FTO* genotypes. This study suggests that in addition to nutritional regulation, *FTO* is also regulated by exercise and may be involved in exercise’s role in reducing obesity risk.

The acute and significant downregulation of *FTO* mRNA following high intensity exercise is a major novel finding of this investigation. A weak trend for genotype by time interaction was also identified suggesting greater downregulation of *FTO* mRNA in the AA genotype compared to the other genotypes (AT and TT). Indeed, AA genotypes demonstrated a 0.32-fold decrease in *FTO* mRNA expression compared to a 0.21-fold and 0.11-fold decrease for AT and TT genotypes respectively, at 10 min post-exercise. Previous studies have shown that *FTO* gene activity is nutritionally regulated with high fat diet, fasting and glucose ingestion all having effects on *FTO* mRNA levels [26–28]. Only one other study has investigated the effect of high intensity training on *FTO* mRNA expression and showed lifestyle changes (diet and exercise) did not impact *FTO* gene expression in peripheral blood mononuclear cells [29]. However, when *FTO* genotype was considered, *FTO* expression was up-regulated in AA genotype carriers and down-regulated in AG/GG genotype carriers in the intervention group [29]. Though the direction of *FTO* mRNA change was opposite to the present study observations, different cell type, age, sex and intervention period, may explain such differences.

The downregulation of *FTO* mRNA following exercise in the present study could be due to its role as an ‘energy sensor’. FTO gene activity is sensitive to the energy status of the cell [30], and it is possible that FTO is responding to the change in energy status and increased energy demand of the muscle as a result of exercise. While a change energy status is quite complex, the current study used an untargeted metabolomics approach to see if the high and/or low intensity bout of exercise could unmask any differences in metabolic responses between *FTO* genotypes that would otherwise not be seen at rest. High intensity exercise induced a significant increase in muscle alanine, erythronate, fumarate, GHB, glucose, glycolate, lactate, malate, maltose, mannose, monopalmitoglycerol and tyrosine during the first 10 min of recovery. LO exercise caused similar but not as many metabolite changes. Despite greater metabolic perturbations following the high intensity compared to the low intensity exercise, O2PLS-DA multivariate regression analysis was unable to distinguish between *FTO* allelic variants based on metabolic profiles following the exercise bouts. A limitation of the O2PLS-DA model is that baseline (resting) and post-exercise measurements are grouped together. By incorporating a time point in which energy demand is at a minimum and under tight homeostatic regulation, the ability to identify differences may be confounded. Indeed, previous investigations have found similar metabolic profiles between allelic variants of *FTO* in at rest [31, 32]. While the current study was unable to identify any specific metabolite(s) and/or metabolic by-product(s), previous research has suggested kreb cycle intermediate fumarate as a potential modifier of FTO. Gerken and colleagues [26] demonstrated inhibition of Fto-catalyzed 1-meA demethylation by fumarate within 2OG decarboxylation assays. While the Gerken study [26] examined FTO function and not gene regulation, it is possible that elevated levels of fumarate (as seen during exercise) are also inhibiting its expression. It is clear that further functional studies are needed to explore other metabolites, especially those significantly impacted by exercise, as possible modifier candidates.

Recent studies have suggested that AMPK may also regulate FTO expression and function in skeletal muscle and could explain another mechanism by which exercise downregulates *FTO* mRNA. Using C2C12 cells, Wu and colleagues [7] showed that inhibition of AMPK upregulates FTO expression and activity and lipid accumulation, while activation of AMPK downregulates FTO expression and activity and reduces lipid accumulation. The current study showed that phosphorylated AMPKα was significantly increased during the early stages of recovery following high intensity exercise, however, no genotype by time interaction was identified. Further, no relationship was found between elevated AMPKα levels and the downregulation of *FTO*. While phosphorylated AMPKα may not be driving the downregulation of *FTO* mRNA in AA genotypes, it could still be impacting the FTO function. Observations from Wu et al. [7] and others [4, 5] suggest that inhibition of FTO function drives higher fat oxidation and lower fat accumulation possibly via FTO-dependent demethylation of mRNA m^6^A. In the current study, fat oxidation and/or markers of lipid accumulation were not measured post-exercise, however, individuals homozygous for the risk A-allele demonstrated greater muscle glucose levels compared to non-risk T-allele at 10 min recovery following both high and low intensity exercise. Higher intramuscular glucose levels post-exercise could reflect a metabolic shift towards greater lipid oxidation and away from glucose oxidation potentially via the AMPK activation and FTO-dependent demethylation of N^6^-methyladenosine mechanism as previously mentioned above. However, acute higher post-exercise intramuscular glucose levels observed in AA genotypes could be due to a number of processes involved in glucose metabolism including glucose delivery and uptake into the muscle, and the resynthesis of glycogen levels post-exercise. Plasma glucose concentrations were measured in the present study and were found to be similar between *FTO* genotypes in response to high intensity exercise and thus it is also unlikely that differences in plasma glucose levels could be responsible for the greater muscle glucose uptake. Glucose uptake into the sarcoplasm depends on the skeletal muscle expression of GLUT4 (an insulin and contraction regulated glucose transport isoform) [33], which is normally increased following exercise and can facilitate post-exercise glucose uptake [34]. Although the current study did not measure GLUT4 translocation directly, we did measure phosphorylation of AS160, an insulin dependent and independent regulator of GLUT4 vesicle movement to, and/or fusion with, the plasma membrane [35]. Despite exercise increasing AS160/Total AS160 in the early stages of recovery, there were no significant differences between genotypes. Though not statistically significant, the AA genotype group did complete their trial on average by about 3–7 min faster and produce higher total workloads in both exercise trials compared to AT and TT genotypes. Further research is needed to determine whether higher intramuscular glucose levels are due to genetic factors inherent in AA genotypes or influences from the aforementioned factors.

Several limitations do exist in the current study. Firstly, we acknowledge that our final sample size (*n* = 28) is relatively low. The average partial eta-squared for observed skeletal muscle variables (VIP metabolites, and protein and mRNA expression levels) was found to be of a medium effect size when performing high and low intensity exercise data analysis (η^2^ = 0.068 and η^2^ = 0.051 respectively). Secondly, we studied both males and females that were young and of “healthy” weight range (BMI range 24–26 kg/m^2^). It is apparent that substrate metabolism is subject to sex-specific regulation. Sex differences in muscle fibre type distribution and substrate availability to, and in, skeletal muscle [36], which also includes molecular differences in glucose and lipid metabolism of skeletal muscle. We used sex as well as age (another known factor) as covariates within our ANCOVA analysis. When sex or age were considered, the reported significant findings were still present. The influence of age was not anticipated given our relatively similar age distribution across alleles. Sex has shown to influence the effect of the *FTO* polymorphism on obesity related traits. However, it was clear from our study that despite sex differences within our cohort (nearly a 50/50 sex split), downregulation of *FTO* mRNA still occurred in the at-risk AA allele and within both sexes. Thirdly, while the *vastus lateralis* muscle is the most common muscle of choice for biopsies because of its accessibility, it is of mixed fibre type composition and thus we cannot comment on fibre type specific differences. Furthermore, it is possible that the sampling window of up to 90 min was insufficient to detect any significant changes in FTO protein content resulting from exercise, with previous research demonstrating changes in the expression levels of other muscle proteins occurring greater than 3 h [37].

## Conclusions

To our knowledge, this is the first study to suggest that disturbed metabolic alterations with exercise, especially those larger perturbations during high intensity exercise, are creating an environment that favours downregulation of *FTO* mRNA. The risk *FTO* allelic variant maybe impacted more, with higher intramuscular glucose levels observed in the AA group compared to AT and TT. We hope that further work extends our preliminary findings to determine if chronic repetitive stimuli (i.e. exercise training), lowers the obesity risk in individuals with the *FTO* risk variant by modulating FTO protein and/or function.

### Legends

Graphpad Prism software 7.02 and SIMCA Statistical Modeling software 14 were used to create artwork.

## Supplementary information

**Additional file 1: Figure S-1** AUC of the ROC curve.

**Additional file 2: Figure S-2** Top 10 Features.

**Additional file 3: Figure S-3** Correlations FTO mRNA and other mRNA.

**Additional file 4: Figure S-4** Correlations Muscle Glucose and mRNA.

**Additional file 5: Figure S-5** Correlations Protein and mRNA.

**Additional file 6: Table S-1** mRNA Assay ID's.

**Additional file 7: Table S-2** Metabolite Parameters.

**Additional file 8: Table S-3** Metabolites (VIP).

## Data Availability

The datasets used and/or analysed during this study are available from the corresponding author on reasonable request.

## Abbreviations

ACTB: Beta-Actin
AMP: Adenosine Monophosphate
AMPK: Adenosine Monophosphate-activated Protein Kinase
AUC: Area Under the Curve
BMI: Body Mass Index
FTO: Fat Mass- and Obesity-Associated Gene
GC-MS: Gas Chromatography-Mass Spectroscopy
GHB: Gamma Hydroxybutyric Acid
GLUT4: Glucose Transporter Type 4
HI: High Intensity Exercise
LO: Low Intensity Exercise
mRNA: Messenger Ribonucleic Acid
mTOR: Mammalian Target of Rapamycin
O2PLS-DA: Orthogonal two Partial Least Squares Discriminant Analysis
RER: Respiratory Exchange Ratio
ROC: Receiver Operating Characteristic
RPE: Ratings of Perceived Exertion
rpm: Revolutions per minute
T. I: Time Interval
VIP: Variable Importance for Projection
W: Workload (watts)
