# Supplementary figures and images for: High intensity exercise downregulates FTO mRNA expression during the early stages of recovery in young males and females
Source: Nutr Metab (Lond). 2020 Aug 17;17:68. doi: 10.1186/s12986-020-00489-1 (PMC7433063; doi:10.1186/s12986-020-00489-1)

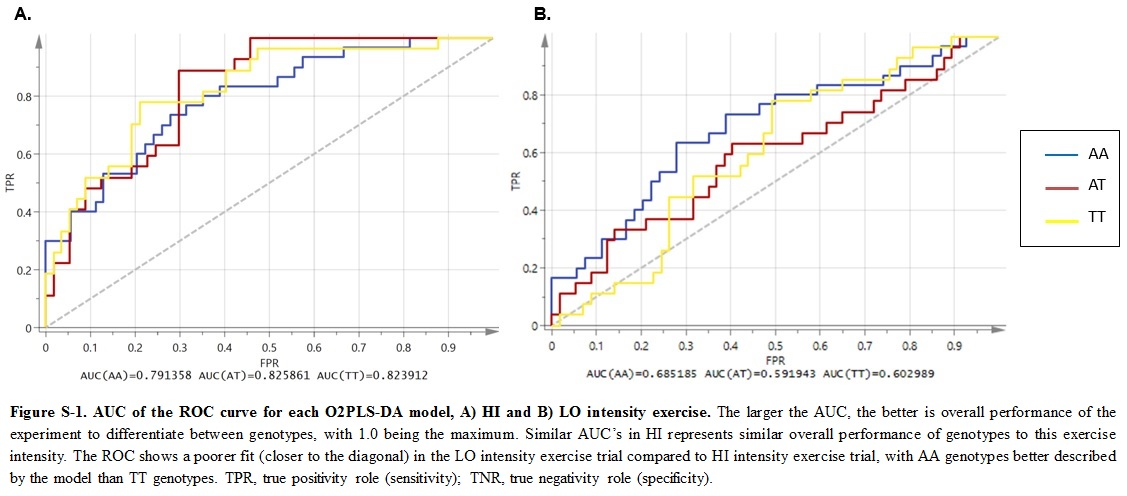

Supplement: Supplementary file 1 — Additional file 1: Figure S-1 AUC of the ROC curve. [file 12986_2020_489_MOESM1_ESM.tiff]

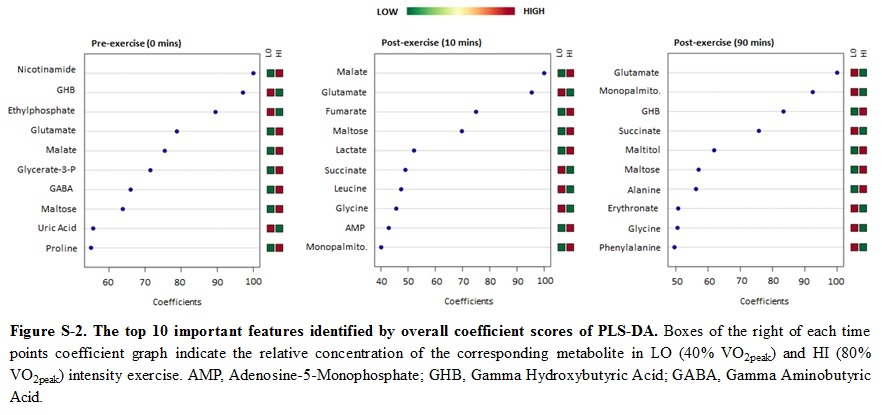

Supplement: Supplementary file 2 — Additional file 2: Figure S-2 Top 10 Features. [file 12986_2020_489_MOESM2_ESM.tiff]

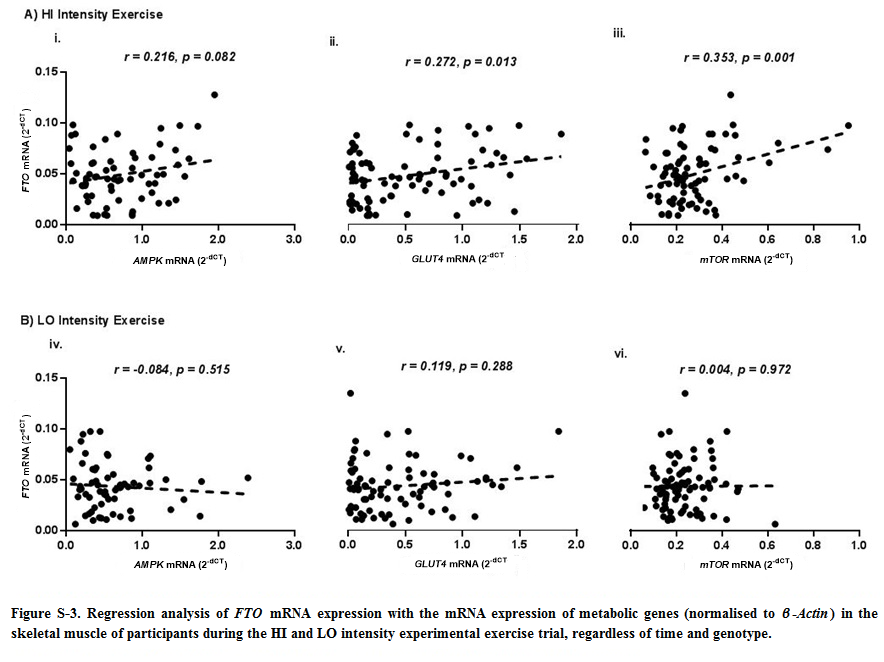

Supplement: Supplementary file 3 — Additional file 3: Figure S-3 Correlations FTO mRNA and other mRNA. [file 12986_2020_489_MOESM3_ESM.tiff]

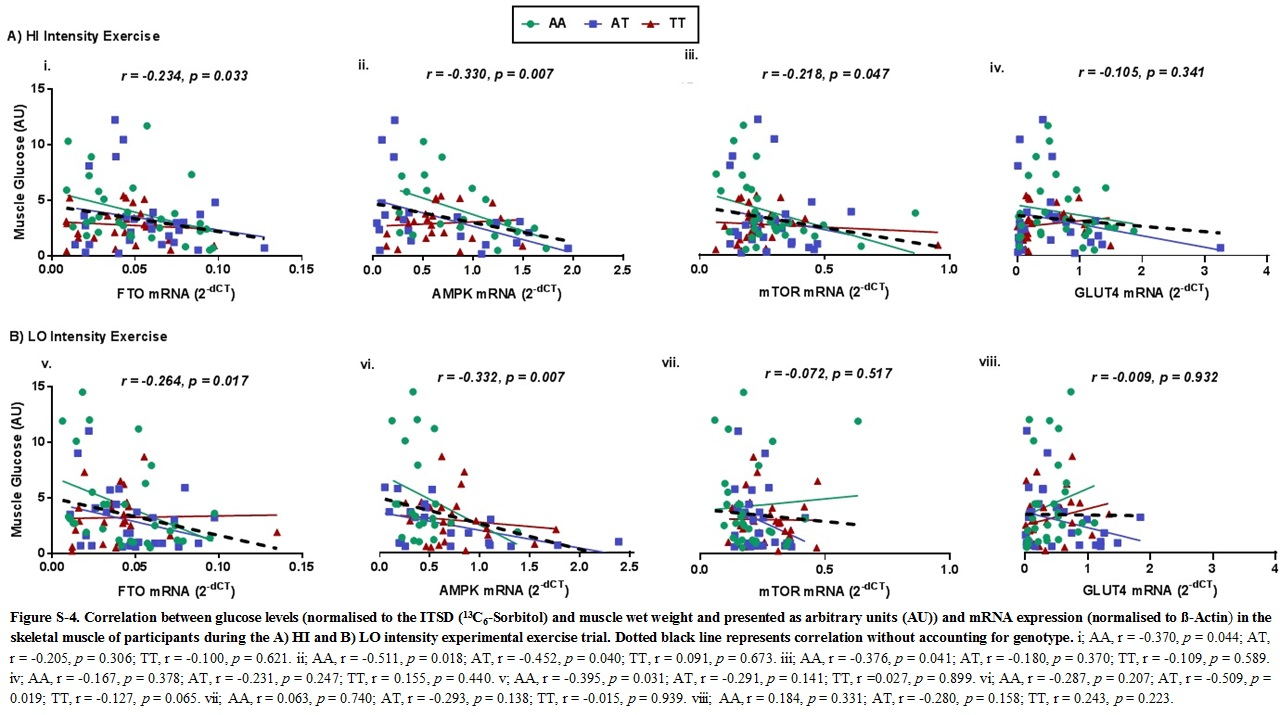

Supplement: Supplementary file 4 — Additional file 4: Figure S-4 Correlations Muscle Glucose and mRNA. [file 12986_2020_489_MOESM4_ESM.tiff]

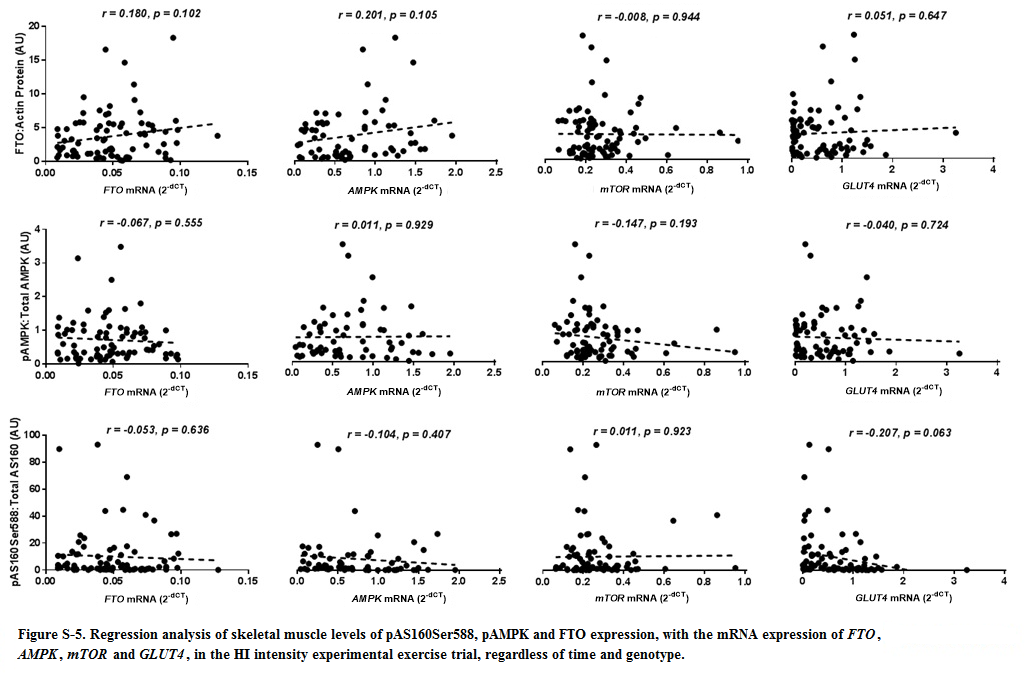

Supplement: Supplementary file 5 — Additional file 5: Figure S-5 Correlations Protein and mRNA. [file 12986_2020_489_MOESM5_ESM.tiff]
